# Salvage low-dose-rate brachytherapy for locally recurrent prostate cancer after definitive irradiation

**DOI:** 10.1016/j.ctro.2024.100809

**Published:** 2024-06-22

**Authors:** Y. Meraouna, P. Blanchard, S. Losa, A. Labib, S. Krhili, P. Pommier, G. Crehange, T. Flam, J-M. Cosset, M. Kissel

**Affiliations:** a Radiotherapy Department, Institut Curie, 26 rue d’Ulm, 75005 Paris, France; b Faculté de médecine Sorbonne Université, 91-105 Boulevard de l’Hôpital, 75013 Paris, France; c Radiotherapy Department, Gustave Roussy, 114 Boulevard Edouard Vaillant, 94220 Villejuif, France; d Physics Department, Institut Curie, 26 rue d’Ulm, 75005 Paris, France; e Urology Department, Clinique Saint Jean de Dieu, 2 rue Rousselet, 75007 Paris, France; f Radiotherapy Department, Centre Charlebourg – La Défense – Amethyst Radiothérapie, 65 Avenue Foch, 92250 La Garenne-Colombes, France

**Keywords:** Prostatic neoplasms, Salvage Therapy, Low dose rate, Brachytherapy, Re-irradiation, Disease-free-survival

## Abstract

**Purpose:**

The optimal management of locally recurrent prostate cancer after definitive irradiation is still unclear but local salvage treatments are gaining interest. A retrospective, single-institution analysis of clinical outcomes and treatment-related toxicity after salvage I-125 low-dose-rate (LDR) brachytherapy (BT) for locally-recurrent prostate cancer was conducted in a Comprehensive Cancer Center.

**Patients and methods:**

A total of 94 patients treated with salvage LDR-BT between 2006 and 2021 were included. The target volume was either the whole-gland +/- a boost on the GTV, the hemigland, or only the GTV. The prescribed dose ranged from 90 to 145 Gy. Toxicity was graded by Common Terminology Criteria for Adverse Events (CTCAE) v5.0.

**Results:**

Median follow-up was 34 months. Initial radiotherapy was external beam radiotherapy in 73 patients (78 %) with a median dose of 76 Gy and I-125 BT in 21 patients (22 %) with a prescribed dose of 145 Gy. Median PSA at salvage was 3.75 ng/ml with a median interval between first and salvage irradiation of 9.4 years. Salvage brachytherapy was associated with androgen deprivation therapy for 32 % of the patients. Only 4 % of the patients were castrate-resistant. Failure free survival was 82 % at 2 years and 66 % at 3 years. The only factors associated with failure-free survival on multivariate analysis were hormonosensitivity at relapse and European Association of Urology (EAU) prognostic group. Late grade 3 urinary and rectal toxicities occurred in 12 % and 1 % of the patients respectively.

No significant difference in toxicity or efficacy was observed between the three implant volume groups.

**Conclusion:**

The efficacy and toxicity results are consistent with those in the LDR group of the MASTER *meta*-analysis. Salvage BT confirms to be an effective and safe option for locally recurrent prostate cancer. A focal approach could be interesting to reduce late severe toxicities, especially urinary.

## Highlights

• 94 patients with isolated local relapse of prostate cancer after definitive irradiation.
• Salvage I-125 low-dose-rate brachytherapy was performed in a Comprehensive Cancer Center.
• Treatment of whole gland (70%) +- with an MRI-based GTV boost (20%) or focal (10%).
• Failure free survival was 66 % at 3 years.
• Late grade 3 urinary and rectal toxicities occurred in 12% and 1% of patients.

## Introduction

Definitive radiotherapy is a standard of care curative treatment option for localized prostate cancer (PCa) in all risk groups. Depending on D’Amico risk classification, External Beam Radiotherapy (EBRT) or Brachytherapy (BT) can be used with or without a combination with androgen deprivation therapy [1].

Despite improvements on the outcomes over the years with advances in diagnostic imaging, RT delivery techniques and dose-escalated radiation, biochemical recurrence (BCR), remains frequent with approximately 10–20 % patients initially treated with radiotherapy experiencing BCR in recent mature phase III studies [2], [3], [4], [5].

While palliative androgen deprivation therapy or observation is often proposed in this setting [6], next generation metabolic imaging techniques such as Choline and especially PSMA PET/CT [7], [8], [9], [10], combined with multiparametric MRI [11], has led to a better detection of isolated local recurrence, eligible for a second local treatment with curative intent.

One of the main challenges in treating patients with radiorecurrent PCa is the risk of significant genitourinary (GU) and gastrointestinal (GI) toxicity associated with local salvage therapies.

Thus, BT which offers delivery of highly conformal high dose radiation with a steep dose gradient and rapid fall off, can minimize dose to organs at risk and hence toxicity [12].

The objective of the study was to report the experience in salvage Low-Dose rate (LDR) BT in terms of efficacy and toxicity.

## Patients and methods

### Patients

Between 2006 and 2021, all consecutive patients treated at Institut Curie, Paris with salvage LDR-BT were included. Eligible patients had received either EBRT or BT, for definitive treatment of localized prostate cancer, at least 2 years prior to salvage BT. The decision to proceed with salvage BT was made after deliberation by the genitourinary Multidisciplinary Tumor Board, and patients received comprehensive information about all the available treatment options. Androgen deprivation therapy was permitted, either as a neo-adjuvant treatment, concurrent with salvage BT or as an adjuvant treatment. Patients under the age of 18, those under legal guardianship, patients who refused to allow their data to be used, or those with histology types other than adenocarcinoma (such as lymphoma or sarcoma) were excluded from the study.

Data for this analysis were obtained retrospectively from the patients' medical records. For each patient, the collected data included demographic information, relevant comorbidities, clinical and technical details of previous cancer and radiation treatments, tumor characteristics at the time of, the presence of any residual side effects from previous radiation therapy, hormone sensitivity or resistance at the time of recurrence, the date of salvage BT, the prescribed BT dose, technical specifics of the procedure, the date of local and distant recurrence, and the patient's last known vital status. We also used the European Association of Urology (EAU) stratification which dichotomizes patients as suitable for salvage treatment or not suitable for salvage treatment. EAU guidelines mention that should be considered for salvage treatment patients with low co-morbidity, a life expectancy of at least 10 years, a pre-salvage PSA < 10 ng/mL and initial biopsy ISUP grade < 2/3, no lymph node involvement or evidence of distant metastatic disease pre-salvage treatment, and those whose initial clinical staging was T1 or T2 [13].

The primary measure of treatment efficacy was the regular monitoring of PSA levels. In cases where hormone therapy was administered concurrently, testosterone levels were also regularly assessed to confirm androgen deprivation. Biochemical recurrence was defined according to Phoenix criteria, which is characterized by a PSA increase of 2 ng/mL or more above the nadir level after treatment [14]. Radiological exams were conducted in instances of PSA relapse or when symptoms suggested possible metastatic progression. The classification of the primary disease was based on the National Comprehensive Cancer Network (NCCN) criteria, with high-risk disease defined by the presence of one or more of the following factors: disease stage ≥ T3a, PSA levels ≥ 20 ng/ml, or a Gleason score ≥ 8 [15]. The severity of treatment-related side effects was reported using the Common Terminology Criteria for Adverse Events (CTCAE) scale, version 5.0 [16], which evaluated urinary toxicity in terms of dysuria, urgency, hematuria, urinary retention, urinary incontinence, and urinary fistula. For digestive toxicity, the criteria included diarrhea, proctitis, rectal hemorrhage, abdominal pain, and rectal fistula. Failure was defined as either biochemical relapse, clinical relapse, or death from any cause.

### Salvage brachytherapy techniques

Pre-salvage BT work-up was dependent on inclusion era and consisted in thoraco-abdomino-pelvic CT scan and bone scintigraphy or Choline PET-CT.

Histological evidence of relapse was obtained whenever possible. The absence of sequelae of previous irradiation was verified by questioning. A cystoscopy and/or rectoscopy was performed if necessary.

BT was delivered in one implant under general anesthesia with real-time transrectal ultrasound (TRUS) guidance. The dose was ranged between 90 and 145 Gy. The target volume was the whole-gland (WG) +/- a boost on the GTV (WG + Boost), the hemigland (HG), or only the GTV (ultrafocal treatment, UF). The patients treated on the whole gland with a boost on the GTV were included in a clinical trial (CAPRICUR, NCT01956058 [17]. All patients during the inclusion period were treated with this method based on CAPRICUR inclusion criteria: localized prostate adenocarcinoma who presented as baseline characteristics a PSA ≤ 20 ng/ml, a Gleason score ≤ 7 and T1c to T2c, who recurred more than 24 months after the end of the first radiotherapy or brachytherapy, PSA at recurrence < 10 ng/ml and who were not started on hormone therapy since the diagnosis of recurrence. Outside from this period, the treated volume was at the discretion of the treating physician. Schematically, patients that had a clear concordance between MRI and biopsies on the localization of the recurrence were selected for a focal or hemigland approach, depending on the size of the index tumor. All other patients (multifocal intraprostatic relapse or discrepancies between MRI and biopsies) were treated on the whole gland.

The needles were implanted at regular intervals in the peripheral prostate in a subcapsular crown pattern. Central needles were then added to essentially cover the apex and base areas. The urethra was defined by the outer surface of the intraprostatic Foley catheter. All structures were outlined on TRUS images. The implants could be performed by the radiation oncologist or by a duo urologist-radiation oncologist. Dose delivered to the different volumes differed within three distinct group of patients according to whether the whole gland, the whole gland + boost, the hemigland or ultrafocal treatment were indicated. Table 2 shows the prescription dose as well as dose-volume constraints to OARs according to this classification. Dose calculation was performed using Variseed v8.0.1 brachytherapy software with real time intra-op module (Varian Medical Systems). Once the dosimetry was validated by the radiation oncologist, Iodine-125 S06 loose seeds (Eckert & Ziegler, Germany) were implanted needle after needle connecting each 15 seed cartridge to a Mick-applicator (Eckert & Ziegler, Germany) under real-time ultrasound (B&K US Systems, Copenhagen, Denmark) guidance according to previous planning. Dosimetry could be manually adjusted in real-time if needed in order to meet dose-volume objectives as well as constraints to OARs. Technical data regarding salvage BT are detailed in Table 3.

**Table 1:** Patients’ characteristics.

	n (%) Median [min − max]	N
Initial Gleason’s score < 8 ≥ 8	79 (84 %) 15 (16 %)	94
Gleason’s score at salvage < 8 ≥ 8	77 (87.5 %) 11 (12.5 %)	88
Initial NCCN risk group Low risk Intermediate High risk / advanced (T3 and/or N1)	20 (21.3 %) 23 (24.5 %) 51 (54.3 %)	94
NCCN risk group (at salvage) Low risk Intermediate High risk / advanced (T3 and/or N1)	16 (18.2 %) 61 (69.3 %) 11 (12.5 %)	88
Interval 1st RT – BT (years)	9.4 [2.2–22.7]	94
Modality of first irradiation EBRT Iodine seeds BT	73 (77.7 %) 21 (22.3 %)	94
Technique of first EBRT 2D 3D IMRT	1 (1.5 %) 47 (69.1 %) 20 (29.4 %)	68
Physical dose of first EBRT (Gy [range])	75 [65–80]	69
Pelvic RT in first radiotherapy	34 (38.2 %)	89
Androgen deprivation therapy associated with first irradiation No Short Long	33 (35.5 %) 51 (54.8 %) 9 (9.7 %)	93
Median PSA at salvage [range]	3.75 [0.41–19]	94
Age at relapse	70 [54–84]	94
Tobacco smoking status Active Former smoker Never smoker	15 (16.3 %) 14 (15.2 %) 63 (68.5 %)	92
History of TURP	3 (3.2 %)	94
Cardiovascular risk factors	58 (61.7 %)	94
History of inflammatory bowel disease	0	94
Performance status at relapse 0 1	68 (78.2 %) 19 (21.8 %)	87
Hormone sensitivity at relapse Hormone sensitive Castrate resistant	90 (95.7 %) 4 (4.3 %)	94
Residual urinary toxicity at relapse Grade 1 Grade 2	42 (45.7 %) 5 (5.4 %)	92
Residual gastro-intestinal toxicity at relapse Grade 1	5 (5.5 %)	91
Residual sexual toxicity at relapse Grade 1 Grade 2 Grade 3	10 (20.8 %) 17 (35.4 %) 9 (18.8 %)	48

NCCN = National Comprehensive Cancer Network; EBRT = External beam radiotherapy; IMRT = Intensity modulated radiotherapy; PSA = prostate specific antigen; TURP = Transurethral resection of the prostate.

**Table 2:** Prescription dose and constraints to OARs from salvage brachytherapy.

Dose-Volume Parameters	WG + Boost	WG	HG/UF
Activity (at implant date in µGy m^2^/h)	0.50	0.509	0.509
Prescription dose (Gy)	90 WG/144 Boost	120	145 (GTV)
D90% (Gy) main volume	90 ≤ D90 ≤ 144	≥ 150	≥ 180
V100 (%)	≥ 99.5 %	≥ 99.5 %	≥ 99.5 %
V150 (%)	≤ 45 %	≤ 50 %	≤ 50 %
V200 (%)	≤ 10 %		
D90% (Gy) Boost	≥ 144		
Urethra D30 (Gy)	< 200		
Urethra D10 (Gy)	< 150		
Urethra D0.1 cc (% of prescribed dose)		≤150 %	≤150 %
Rectum D2cc (% of prescribed dose)		≤100 %	≤100 %

WG = Whole Gland; HG = Hemigland; UF = Ultrafocal.

**Table 3:** Technical data from salvage brachytherapy.

	All patients	WG + Boost	WG	HG/UF
Characteristic	Median [min – max]			
CTV volume (cc)	18.1 [4.9 – 50.7]	20.7 [11.6–28.7]	18.1 [7.1–50.7]	7.3 [4.9–17.4]
Number of seeds	39 [18 – 71]	40 [25–50]	40 [25–71]	24 [18–45]
D90 (% of prescribed dose)	129.1 [116.0 – 221.9]	155 [116–222]	128 [121–179]	126 [121–132]
D100 (% of prescribed dose)	97.3 [64.7 – 119.5]	104 [65–119]	96 [75–113]	86 [73–100]
V100	100.0 [93.8 – 100]	100 [94–100]	100 [97–100]	100 [98–100]
V150	63.6 [36.4 – 97.0]	92 [36–97]	61 [51–92]	65 [58–74]
V200	25.1 [17.0 – 68.7]	56 [30–69]	24 [17–51]	25 [18–38]
Urethra D0.1 cc (% of prescribed dose)	137.0 [90.8 – 204.1]	177 [148–204]	135 [121–179]	108 [91–138]
Rectum D0.1 cc (% of prescribed dose)	90.2 [26.1 – 167.2]	126 [86–158]	86 [26–167]	84 [60–121]
Rectum D2cc (% of prescribed dose)	54.5 [16.8 – 94.2]	79 [48–94]	52 [17–83]	46 [32–63]

WG = Whole Gland; HG = Hemigland; UF = Ultrafocal.

The patients were discharged the morning following the procedure.

### Ethics

This retrospective study was institutional review-board–approved. A specific information note was sent to patients.

### Statistics

Qualitative variables were described by using numbers and percentages, and quantitative variables by using mean (+/- standard deviation) or median and range in case of non-normal distribution. Overall survival, local and distant recurrence-free survival curves were estimated by the Kaplan-Meier method and calculated from the date of salvage BT. The median time and survival rates at different points since the treatment start were estimated with their 95 % confidence interval. The log-rank test and the Cox model were used to compare survival curves according to observed characteristics. The effect of continuous variables on survival was evaluated both continuously and through a log-rank test.

Statistical analyses were performed using SAS software v9.4.

## Results

A total of 94 patients were included. Patients’ characteristics are presented in Table 1. All patients but one had pathologically confirmed relapse (92/93). Pre BT work up consisted in Choline PET/CT for 93 % of patients (followed by PET-PSMA for 1 patient), conventional CT + bone scintigraphy for 4 % of patients and prostatic MRI for 98 % of patients. All patients were node negative on pre-BT work up.

Eighteen patients (19 %) underwent a rectoscopy and twelve patients a cystoscopy (13 %) prior to salvage BT to check the absence of residual radiation rectitis/cystitis. None of the patients had a grade 3 or higher urinary or GI toxicity after primary radiotherapy.

**Fig. 1:**
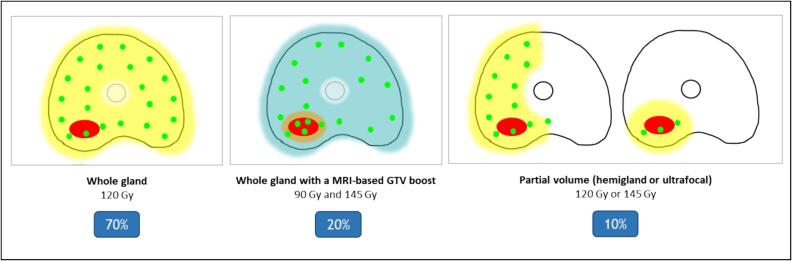
Schematic representation of the different implant volume groups.

Salvage BT implant volume was WG (70 %) with a dose prescription of 120 Gy for a large majority of patients; WG + boost (20 %) with a dose prescription of 90 Gy to the whole gland and 144 Gy to an MRI-based GTV; or a partial volume (HG or UF) (10 %) with a dose prescription of 120 Gy (or 145 Gy for 1 patient only) (Fig. 1). Salvage BT was associated with a short (6 months) hormone deprivation therapy for 32 % of the patients.

### Efficacy

**Fig. 2:**
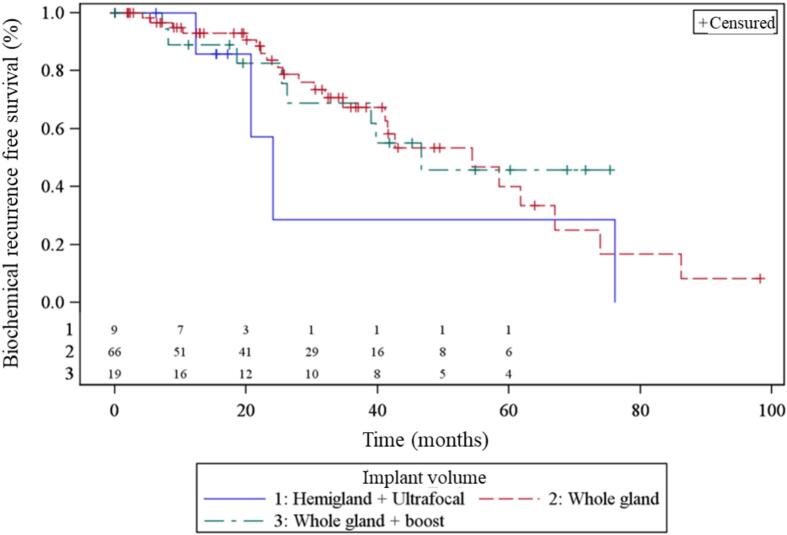
Progression free survival according to implant volume.

With a median follow-up of 34.3 months (range: 0–––182.2 months), 32 % of patients had a biochemical relapse, 21 % of patients presented with a 2nd local relapse, 13 % a nodal relapse and 9 % a distant relapse. Median PSA nadir was 0.11 ng/ml [Q1 – Q3: 0.02–––0.37]. Failure-free survival was 82 % at 2 years and 66 % at 3 years in the entire population. No significant difference in efficacy was observed between the three implant volume groups (Fig. 2).

On univariate analysis, castration resistance at relapse was a negative prognostic factor.

EAU prognostic group and Gleason at relapse were significantly associated with bPFS on univariate analysis in hormone sensitive patients only. On multivariate analysis (Table 4), hormonosensitivity at relapse and EAU prognostic group were the only two factors significantly associated with biochemical progression free survival (bPFS).

**Table 4:** Univariate and multivariate analyses for biochemical progression free survival.

Patients’ characteristics		Univariate		Multivariate	
		HR (95 % CI)	*p*	HR (95 % CI)	*p*
EAU prognostic group	Adequate for salvage RP	1	0.0849	1	0.0266
	Not adequate for salvage RP	2.07 (0.91; 4.73)		3.22 (1.25; 8.28)	
Time between first RT and salvage BT	> 10y	1	0.5652	1	0.0807
	5–10y	0.82 (0.41; 1.63)		0.47 (0.20; 1.11)	
Volume of implant	Whole gland + boost	1		1	0.1993
	Hemigland + Ultrafocal	1.98 (0.57; 6.88)	0.2807	2.87 (0.76; 10.93)	
	Whole gland	1.08 (0.48; 2.44)	0.8557	0.66 (0.27; 1.64)	
Hormone sensitivity at relapse	Yes	1	**0.0026**	1	**0.0007**
	No	5.51 (1.82; 16.71)		15.67 (3.81; 64.50)	
Gleason score at relapse	≤7	1	0.1077		
	8–10	2.11 (0.85; 5.24)			
PSA at relapse	< 10 ng/mL	1	0.1842		
	> 10 ng/mL	2.26 (0.68; 7.51)			
Initial high risk	No	1	0.3743		
	Yes	1.36 (0.69; 2.69)			

HR = Hazard Ratio; CI = Confidence Interval; EAU = European Association of Eurology; RP = Radical Prostatectomy; BT = Brachytherapy; PSA = Prostate Specific Antigen.

After relapse, 70 % of patients received a first-generation androgen deprivation therapy, 10 % a 3rd local treatment and one patient received a second-generation androgen deprivation therapy.

During follow-up, 7 patients died, 2 from prostate cancer, 5 from other causes (two from other neoplasms, none of which were in the pelvis, one from unknown cause).

### Toxicity

Few immediate complications of salvage BT were reported: three patients had a urinary infection (Grade 2), five patients suffered from urinary retention (two Grade 1 and three Grade 2) and six patients presented with hematuria (five Grade 1 and one Grade 2).

Acute grade 2 urinary and gastro-intestinal toxicities were reported in 46.1 % and 2.2 % of patients respectively. No Grade 3 + acute toxicities were reported.

Late Grade 2 + urinary and gastro-intestinal toxicity was reported in 63.0 % and 6.2 % of patients respectively, including ten (12.3 %) and one (1.2 %) Grade 3 toxicities (Table 5). No late Grade 4 toxicity nor fistula were reported. The most reported urinary symptoms were chronic retention (43.2 % Grade 2 + ) and frequency (30.9 % Grade 2). During follow-up, 13 patients had to undergo invasive procedures for late toxicity: three patients had a urinary catheter for clot removal (hematuria), five patients had a ureteral dilation, and eight patients had a *trans*-urethral prostate resection.

**Table 5:** Acute and late toxicities.

	Grade 1	Grade 2	Grade 3	Grades 4–5
Acute GU	66 (74 %)	41 (66 %)	0	0
Acute GI	18 (20 %)	2 (2 %)	0	0
Late GU	25 (31 %)	41 (51 %)	10 (12 %)	0
Late GI	26 (32 %)	4 (5 %)	1 (1 %)	0

GU = genito-urinary; GI = gastro-intestinal.

No statistically significant difference was observed in late toxicity between implant volume groups. Late Grade 2 + urinary toxicity occurred in 36/66 patients with whole gland implantation (54.5 %), 11/19 patients with whole gland implantation with a boost (57.9 %) and 4/9 patients with partial implantation (hemigland or ultrafocal) (44.4 %).

All late Grade 2 + gastro-intestinal toxicities occurred in patients with whole gland implantation.

## Discussion

The two-year bPFS in our study was 82 % with severe late GU/GI toxicity rates of 12 % and 1 % respectively, comparable to previously published reports of salvage LDR BT (Table 6), showing that this is a reasonable therapeutic option in this setting.

**Table 6:** Literature review of salvage low dose rate brachytherapy.

Author Year	N	Design	Proportion of high risk	Median PSA at relapse (ng/ml) [range]	Prescription dose (Gy)	Treated volume	ADT use	Staging method at relapse	bPFS	G3 + late GU tox	G3 + late GI tox
Grado 1999 [55]	49	Retrospective	NR	5.6 [1.5–79.1]	160	WG	16 %	Bone scan + CT	48 % 3y 34 % 5y	14 %	2 %
Beyer 1999 [56]	17	Retrospective	NR	2.2 [0.3–27]	120/90	WG	47 %	Bone scan +/- CT (8/17)	53 % 5y	NR	NR
Koutrouvelis 2003 [57]	31	Retrospective	NR	NR	100–120 (103Pd) / 120–144 (125I)	WG +/- SV	97 %	NR	87 %	13 %	6 %
Wong 2006 [58]	17	Retrospective	NR	4.7 [1.2–11.8]	126	WG	100 %	NR	75 % 4y	47 %	6 %
Nguyen 2007 [47]	25	Prospective	0 %	5.5 [1.4–11.6]	137	Partial	0 %	Pelvic CT or MRI + Bone scan	70 % 4y	20 %	20 %
Lee 2008 [59]	21	Retrospective	NR	3.8	90	WG	57 %	Bone scan + CT	94 % 3y 38 % 5y	0 %	0 %
Aaronson 2009 [60]	24	Retrospective	NR	3.41 [0.3–10]	144 PTV/108 WG	WG + Boost	17 %	Bone scan + MRI	89 % 3y	4 %	0 %
Burri 2010 [34]	37	Retrospective	24 %	5.6 [1.7–35.0]	110 (103Pd) / 135 (125I)	WG	83.7 %	CT + Bone scan	65 % 5y 54 % 10y	11 %	0 %
Moman 2010 [36]	31	Retrospective	NR	11.4 (+/- 7.6) (mean +/- SD)	145	WG	16.1 %	CT + Bone scan	51 % 1y 20 % 5y	20 %	6 %
Hsu 2013 [25]	15	Retrospective	0 %	3.5 [0.9–5.6]	144	Partial	0 %	CT + bone scan	71 % 3y	0 %	0 %
Vargas 2013 [61]	69	Retrospective	NR	NR	100	WG	89.9 %	CT + Bone scan	67 % 5y	8.7 %	0 %
Henriquez 2014 [35]	56 (37 LDR)	Retrospective	28.5 %	3.7 [1.1–30]	145 (120–160)	WG	16 %	NR	77 % 5y	24 %	2.7 %
Peters 2014 [26]	20	Retrospective	60 %	4.7 [0.3–14]	≥ 144	Partial	40 %	Choline-PET (50 %)	60 % 5y	5 %	0 %
Lacy 2016 [62]	21	Retrospective	0 %	6.3 [1–19]	108–144	Partial	10 %	CT + bone scan	60 % 3y	14 %	5 %
Peters 2016 [37]	62	Retrospective	NR	8.6 [0.1–92.6]	145	WG	34 %	Bone scan + CT or MRI	46 % 3y 28 % 5y	30 %	8 %
Kunogi 2016 [27]	12	Retrospective	0 %	4.1 [2.9–8.2]	145	Partial	25 %	CT + bone scan	78 % 4y	0 %	0 %
Kollmeier 2017 [38]	98 (37 LDR)	Retrospective	NR	3.7 [0–59]	125 (103Pd) / 144 (125I)	WG	46 %	NR	60.2 %	2.7 %	0 %
Smith 2020 [33]	108	Retrospective	NR	5.3 [0.1–38.4]	100 (80–124)	WG	94 %	CT + Bone scan +/- MRI +/- PET	63.1 % 5y 52 % 10y	15.7 %	2.8 %
Pons-Llanas 2020 [63]	30	Retrospective	20 %	3.5 [2.86–5.74]	120–130	WG	20 %	Choline PET-CT + MRI	86.7 % 1y 56.7 % 3y 53.3 % 5y	16.7 %	3.3 %
Crook 2021 [22], [23]	92	Prospective	0 %	7.3 [5.5–9.3]	140/120	WG (2 pts partial)	16 %	Bone scan + CT	54 % 10y ∼80 % 2y	12.6 %	1.1 %
Present study	94	Retrospective	54.3 %	3.75 [0.41–19]	120; 90/145	WG (70 %) WG + Boost (20 %) Partial (10 %)	32 %	Choline PET-CT + MRI	82 % 2y 66 % 3y	12 %	1 %

PSA = Prostate specific antigen; GU = Genito-urinary; GI = Gastro-intestinal; ADT = Androgen deprivation therapy; bPFS = Biochemical progression free survival; NR = Not reported; SD = Standard derivation; WG = Whole gland; SV = Seminal Vesicles; MRI = magnetic resonance imaging.

Patients with a rising PSA after definitive radiotherapy for localized prostate cancer should undergo comprehensive imaging assessment and those with no distant disease and biopsy proven local recurrence in the prostate may be eligible to local salvage therapy. While multiple salvage treatment options are available, a significant number of patients in this context are often offered lifelong androgen deprivation therapy, which is purely palliative in nature and comes with substantial morbidity [18], [19]. Data from the Spanish Registry of Prostate Cancer (RECAP) revealed in 2016 that over 70 % of patients who experienced biochemical failure (BF) following radiotherapy received ADT, while only 6 % received salvage BT [20].

A systematic review and *meta*-analysis comparing the efficacy and toxicity of all possible salvage treatments (salvage RP, salvage HIFU, salvage cryotherapy, SBRT, salvage LDR brachytherapy, and salvage HDR brachytherapy) in patients with locally recurrent prostate cancer after primary definitive radiation therapy, found no significant differences in Recurrence Free Survival (RFS) at 5 years between RP and other salvage modalities [21]. The severe GU toxicity rates were highest for HIFU and RP, exceeding 21 % and to a lesser extent for Cryotherapy with 15 %, whereas it ranged from 5.6 % to 9.6 % with re-irradiation. Severe GI toxicity rates were relatively low with all modalities, ranging from 0 % to 2.1 %, seemingly favoring HDR-brachytherapy. In this *meta*-analysis, two year and five-year PFS in LDR-BT studies were 79 % and 53 % respectively, while severe (≥grade 3) genitourinary toxicity rate was 9.1 %, significantly lower than the 21 % rate observed in RP studies (p = 0.001).

The results presented in our study are consistent with other salvage LDR BT studies, for example in the prospective phase II trial RTOG/NRG 0526, 2-year bPFS was approximately 80 % (82 % in present study) with 14 % late GU/GI toxicity (13 % in present study). With a longer median follow-up of 6.7 years, DFS was 61 % and 33 % at 5 and 10 years respectively with a 10-year bPFS of 54 % [22], [23]. However, the study only included patients with low to intermediate initial risk, which was not the case in our cohort (54 % of initial high risk) and toxicity is probably underestimated in our study due to its retrospective nature. Interestingly, the only factor predictive of severe late adverse events in this study was the volume of prostate that receives 100 % of the prescription dose (V100), suggesting that partial salvage therapy may improve toxicity outcomes, while it was not found in our study.

Of note, the present study is to our knowledge the largest study evaluating patients with partial as well as whole gland LDR salvage BT. However, this study failed to demonstrate significant improvement in toxicity with a partial implantation volume, although no patient with ultrafocal or hemigland implantation had grade 3 or more GU/GI toxicity. This may be due to a lack of power given the relatively small subset of patients receiving focal salvage BT in our study. Indeed, a narrative review on focal versus whole gland salvage BT conducted by the MD Anderson found lower median rate of grade 3 or higher GU toxicity (4 % vs 12 %) and GI toxicity (0 vs 3 %) with focal compared to WG salvage BT [24]. For example, Hsu et al evaluated feasibility and clinical outcomes of partial salvage LDR-BT and found minimal treatment-related toxicity, with no grade 3 GI/GU toxicity, and a local control comparable to conventional salvage with 3 year PFS of 71.4 % [25]. Similarly, Peters et al reported a 60 % 3-year bPFS with only one G3 GU toxicity (urethral stricture) in 20 patients treated with focal multiparametric MRI-TRUS guided I125 salvage [26]. Another small retrospective study of focal partial salvage LDR BT showed encouraging results with a 4-years bPFS of 78 % and no grade 3 + GU/GI toxicity [27]. More studies of focal salvage HDR BT reported very low rates of grade 3 toxicity [28], [29], [30].

The MASTER *meta*-analysis reported that salvage SBRT (sSBRT) had the lowest severe toxicity rate of all salvage treatment options with comparable RFS [21]. Nevertheless, the number of studies encompassing sSBRT in the *meta*-analysis was notably limited, and the median follow-up was relatively short at 26 months. Recently, a retrospective study with a longer median follow-up of 38.6 months showed a high incidence of grade ≥ 3 toxicity (32.1 % of patients) [31], statistically significantly different between focal (15 patients) and whole-gland (41 patients) sSBRT in favor of the focal treatment group (43.8 % vs. 7.1 % at 2 years; p = 0.006). The size of the Planning Target Volume (PTV) was an independent prognostic factor of the occurrence of grade 3 + toxicity. Moreover, no significant difference was observed in local control, PFS or bPFS between the two volume groups.

Early results of focal sSBRT series are promising with for example a reported late grade 2 GU toxicity rate of only 4 % (no grade 3 toxicity except for one G3 GI acute toxicity) in a prospective study on ^68^Ga-PSMA PET/CT and MRI guided focal sSBRT, with a two-years bPFS of 80 % [32].

The only prognostic factors significantly associated with bPFS on the multivariate analysis in our study were hormonosensitivity at relapse and EAU prognostic group. Nevertheless, other studies regarding LDR BT in this setting found initial PSA [33], PSA at salvage [34], [35], PSA doubling time (DT) [36], [37], [38], ISUP score [33], [36] and disease-free interval following primary therapy [35], [37], to be prognostic factors associated with biochemical relapse-free survival.

In a Delphi consensus on salvage BT by the Uro-GEC group of GEC-ESTRO (Groupe Européen de Curiethérapie – European Society for Radiotherapy and Oncology), international experts collectively agreed upon following selection criteria (level of agreement expressed as percentage): ECOG/WHO performance score of 0 or 1 (89 %), ≤ T3b both at primary and at time of relapse (81 %), Gleason score at primary treatment ≤ 8 (95 %), maximum of International Prostate Score Symptom (IPSS) from 8 to 15 (88 %), 12–24 biopsies should be performed at relapse (83 %) [39].

The prognostic value of the pre-salvage biopsy Gleason score (GS) remains debatable. Indeed, post-irradiation Gleason score grading is challenging due to the well described effects of radiotherapy on the histological appearance of tumor and non-tumor prostatic tissue. A study comparing pathological results from biopsy and surgery in patients undergoing salvage radical prostatectomy found that 58 % of patients had upgrading of their pathology after surgery [40]. However, pre-salvage biopsy GS was a strong prognostic risk factor for progression free survival in a systematic review of salvage radical prostatectomy and thus seemed relevant to include in the analysis [41].

In a more recent Delphi consensus published in 2023, thirty international experts achieved consensus on several areas of patient selection, including: a minimum of 2–3 years from initial RT to salvage BT and that MRI and PSMA PET should be obtained prior to reirradiation [42]. In both studies, no consensus was obtained regarding the optimal dose schedule or treatment volume. In our cohort, no dose–effect relationship could be identified but patients receiving different doses had different implant volumes making any comparison difficult. Well conducted prospective studies are needed to answer remaining questions regarding type and technique of salvage BT.

Our study has some weaknesses. Firstly, the subdivision of our patients into three implant volume groups (whole gland, whole gland + boost or partial gland) aimed to help define the best treatment option but this in turn introduces a degree of heterogeneity into the cohort. Secondly, most patients in our study were staged using MRI and Choline PET/CT which compare favorably to conventional imaging with CT scan and bone scan but may have missed low-volume metastasis which can be diagnosed by novel PET imaging with prostate-specific radio pharmaceutics. Thirdly, due to the relatively short follow-up, late toxicities may have been underestimated.

## Conclusion

Salvage BT surely is gaining interest for radiorecurrent prostate cancer [43], [44], [45], [46], [47], [48], [49]. The present study as well as current literature validate salvage LDR brachytherapy as an interesting option for patients with isolated local recurrence of prostate cancer after definitive radiation therapy, with a toxicity profile comparing favorably with other local salvage options and potential for long-term disease control. However, these findings rely on essentially retrospective and relatively small studies which provides poor evidence level, and the results of ongoing prospective studies are awaited. Furthermore, the non-negligible risk of severe toxicity after salvage BT and especially late urinary toxicity remains a concern. Many ongoing clinical trials aim to evaluate further focal salvage BT with potentially less adverse effects, such as F-Sharp (NCT03312972) [50], FocaSaBra (NCT05715502) [51], PROSALBRA [52], or the SalvageHDR study (NCT03246802) and a phase I/II trial (NCT01583920) [53], [54]. In the meantime, salvage brachytherapy should be performed on carefully selected patients in experienced centers ideally within randomized clinical trials or prospective registry studies.

## Patient consent statement

This retrospective chart review study involving human participants was in accordance with the ethical standards of the institutional and national research committee and with the 1964 Helsinki Declaration and its later amendments or comparable ethical standards. The Human Investigation Committee (IRB) of Institut Curie approved this study.

## CRediT authorship contribution statement

**Y. Meraouna:** Investigation, Data curation, Writing – original draft, Visualization. **P. Blanchard:** Conceptualization, Methodology, Formal analysis, Writing – review & editing, Visualization. **S. Losa:** Writing – review & editing. **A. Labib:** Writing – review & editing. **S. Krhili:** Writing – review & editing. **P. Pommier:** Writing – review & editing. **G. Crehange:** Writing – review & editing. **T. Flam:** Writing – review & editing. **J-M. Cosset:** Writing – review & editing. **M. Kissel:** Conceptualization, Methodology, Resources, Writing – review & editing, Supervision, Project administration.

## Declaration of competing interest

The authors declare that they have no known competing financial interests or personal relationships that could have appeared to influence the work reported in this paper.
